# Comparative proteomic analysis of *Salmonella enterica *serovar Typhimurium ppGpp-deficient mutant to identify a novel virulence protein required for intracellular survival in macrophages

**DOI:** 10.1186/1471-2180-10-324

**Published:** 2010-12-21

**Authors:** Takeshi Haneda, Mariko Sugimoto, Yukie Yoshida-Ohta, Yoshio Kodera, Masamichi Oh-Ishi, Tadakazu Maeda, Satomi Shimizu-Izumi, Tsuyoshi Miki, Yoshinori Kumagai, Hirofumi Danbara, Nobuhiko Okada

**Affiliations:** 1 Department of Microbiology, School of Pharmacy, Kitasato University, 5-9-1 Shirokane, Minato-ku, Tokyo 108-8641, Japan; 2 Laboratory of Biomolecular Dynamics, Department of Physics, Kitasato University School of Science, 1-15-1 Kitasato, Minami-ku, Sagamihara City, Kanagawa 252-0373, Japan

## Abstract

**Background:**

The global ppGpp-mediated stringent response in pathogenic bacteria plays an important role in the pathogenesis of bacterial infections. In *Salmonella enterica *serovar Typhimurium (*S*. Typhimurium), several genes, including virulence genes, are regulated by ppGpp when bacteria are under the stringent response. To understand the control of virulence genes by ppGpp in *S*. Typhimurium, agarose 2-dimensional electrophoresis (2-DE) combined with mass spectrometry was used and a comprehensive 2-DE reference map of amino acid-starved *S*. Typhimurium strain SH100, a derivative of ATCC 14028, was established.

**Results:**

Of the 366 examined spots, 269 proteins were successfully identified. The comparative analysis of the wild-type and ppGpp^0 ^mutant strains revealed 55 proteins, the expression patterns of which were affected by ppGpp. Using a mouse infection model, we further identified a novel virulence-associated factor, STM3169, from the ppGpp-regulated and *Salmonella*-specific proteins. In addition, *Salmonella *strains carrying mutations in the gene encoding STM3169 showed growth defects and impaired growth within macrophage-like RAW264.7 cells. Furthermore, we found that expression of *stm3169 *was controlled by ppGpp and SsrB, a response regulator of the two-component system located on *Salmonella *pathogenicity island 2.

**Conclusions:**

A proteomic approach using a 2-DE reference map can prove a powerful tool for analyzing virulence factors and the regulatory network involved in *Salmonella *pathogenesis. Our results also provide evidence of a global response mediated by ppGpp in *S. enterica*.

## Background

The facultative intracellular bacterium *Salmonella enterica *causes a broad spectrum of diseases, such as gastroenteritis and bacteremia, which are typically acquired by oral ingestion of contaminated food or water. *S. enterica *serovar Typhimurium (*S*. Typhimurium) causes enterocolitis in humans and a typhoid-like systemic infection in mice.

Several virulence genes associated with *Salmonella *pathogenicity islands (SPIs) and the virulence plasmid have been characterized in *S*. Typhimurium. Two type III secretion systems (T3SS) encoded by SPI-1 and SPI-2 play central roles in *Salmonella *pathogenesis. SPI-1 is essential for the invasion of host cells and the induction of apoptosis in infected macrophages [1,2]. SPI-2 T3SS primarily confers survival and replication on macrophages and is required for systemic infection in the mouse infection model [3,4]. Expression of SPI-2 genes is induced within a modified phagosome, called the *Salmonella*-containing vacuole (SCV), in infected macrophages [5]. Induction of SPI-2 genes depends on a two-component regulatory system, SsrA/SsrB, encoded within the SPI-2 region [6]. Expression of SsrAB is also mediated by two-component regulatory systems, OmpR/EnvZ and PhoP/PhoQ, which sense osmotic stress and cation limitation, respectively [7,8]. In addition, a global transcriptional regulator, SlyA, which interacts directly with the *ssrA *promoter region, is involved in the expression of SPI-2 T3SS [9-11].

During infection of mammalian hosts, *S*. Typhimurium has to rapidly adapt to different environmental conditions encountered in its passage through the gastrointestinal tract and its subsequent uptake into epithelial cells and macrophages. Thus, establishment of infection within a host requires coordinated expression of a large number of virulence genes necessary for the adaptation between extracellular and intracellular phases of infection. It has been demonstrated that the stringent response plays an important role in the expression of *Salmonella *virulence genes during infection [12-14].

The stringent response is mediated by the signal molecules, guanosine tetraphosphate (ppGpp) and guanosine pentaphosphate (pppGpp) (both are referred to as ppGpp in this manuscript), which accumulate in bacterial cells and exert both positive and negative effects on the transcription of many genes. ppGpp plays an important role in the virulence of pathogenic bacteria [15]. In Gram-negative bacteria, ppGpp is synthesized by two tynthases, the synthase I and the synthase II, which are encoded by the *relA *and *spoT *genes, respectively [16]. These enzymes respond differently to environmental conditions. RelA is activated by the binding of uncharged tRNA to ribosomes upon amino acid starvation. SpoT is induced during the exponential growth phase and responds to other changes in environmental conditions, specifically a lack of carbon sources or energy deprivation [17]. ppGpp binds directly to the β and β' subunits of RNA polymerase (RNAP), leading to destabilization of the RNAP-rRNA promoter open complex [18]. Moreover, the stringent response is increased by the availability of free RNAP, which gives rise to σ competition [19]. ppGpp indirectly activates the expression of many stress-induced genes by its release from RNAP σ^70^-dependent promoters and by facilitating the use of alternativeσ factors. It has been shown that ppGpp is not only essential for surviving periods of stress but also for the interaction of bacteria with their host [20].

In case of *S*. Typhimurium, a mutant strain deficient in both *relA *and *spoT *(Δ*relA*Δ*spoT*) shows marked reductions in both bacterial invasion into host cells and proliferation in macrophages [12,13]. Furthermore, the virulence of the Δ*relA*Δ*spoT *mutant is severely attenuated in mice [12,13]. ppGpp controls the expression of SPI-1 to -5 and Spv through their transcriptional regulators HilA, InvF, RtsA, SsrA, SlyA, and SpvR [12-14,21]. These observations indicate that ppGpp may play a major role in *Salmonella *virulence via the altered expression of regulatory genes. Because ppGpp has been shown to affect the expression of many virulence genes in *S*. Typhimurium, it is likely that there are additional virulence genes among the ppGpp-regulated genes.

In this study, we constructed an agarose 2-dimensional electrophoresis (2-DE) reference map of *S*. Typhimurium grown under amino acid starvation to identify ppGpp-regulated proteins from whole-cell preparations. By comparative proteomic analysis of ppGpp-regulated and *Salmonella-*specific proteins, we identified a novel virulence factor, STM3169, required for intracellular survival within macrophages.

## Results and Discussion

### Agarose 2-DE reference map of *S*. Typhimurium with induced stringent responses

**Figure 1:**
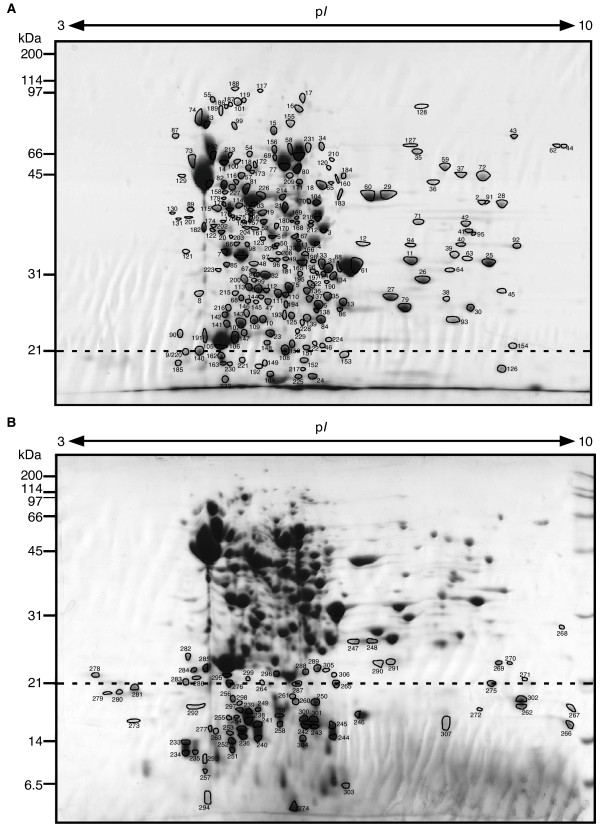
**Agarose 2-DE reference map of the *S*. Typhimurium strain SH100, prepared using a 12% gel focused on high-molecular-mass proteins (A) and a 15% gel focused on low-molecular-mass proteins (B)**. Strain SH100 was grown under amino acid starvation as described previously [26]. Gels are stained with Coomassie Brilliant Blue. Identified spots are numbered (corresponding to the spot numbers in additional file: 1. Proteins identified on the reference map).

Because the correlation between mRNA and protein expression levels is nonpredictive, the direct measurement of protein expression is essential for the analysis of biological processes [22]. 2-DE allows several hundred proteins to be displayed on a single gel, thus producing a direct and global view of the proteome at a given time point [23]. Agarose 2-DE takes advantage of the process of protein separation over a broad range [24,25]. In this study, to separate and identify more proteins, we applied agarose 2-DE to the bacterial proteome, and also used 12% and 15% SDS-PAGE gels for the second dimension. Whole-cell proteins were obtained from the *S*. Typhimurium strain SH100, a derivative of ATCC 14028, with the stringent response induced by serine hydroxamate, as described previously [26]. Agarose 2-DE was performed at least three times on independent samples. More than 350 protein spots from the strain were detected on each 2-DE gel stained with Coomassie Brilliant Blue. To identify proteins on the agarose 2-DE gels, we excised 230 spots from the 12% gel and 136 spots from the 15% gel. We finally identified a total of 360 proteins (273 proteins from the 12% gel [Figure 1A] and 87 proteins from the 15% gel [Figure 1B]) by MS/MS analysis out of 307 protein spots (232 spots from the 12% gel and 75 spots from the 15% gel) that were successfully excised (see additional file: 1). In total, 267 proteins were obtained from the gels, with 40 proteins identified as being redundant. The highest and lowest molecular masses of identified proteins were 93.4 kDa for AcnB (aconitate hydrase 2, spot 188) and 7.4 kDa for CspC (cold-shock protein, spot 303), respectively. Fifty spots (35 spots from the 12% gel and 15 spots from the 15% gel) were found in a basic range. Interestingly, 78 protein spots (25.4%) were annotated as putative proteins on the genome of the *S*. Typhimurium LT2 strain, which is more than 98% identical in sequence to the 14028 strain [27].

**Figure 2:**
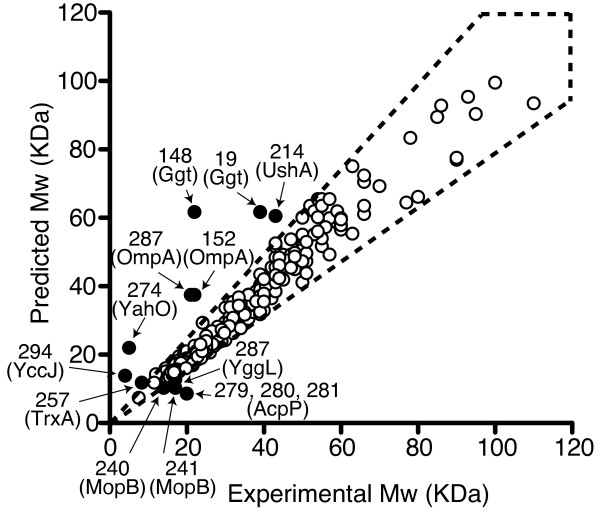
**Comparison of the gel-estimated and theoretically calculated molecular weight (Mw) of the identified protein spots**. Arrows indicate spot numbers and identified proteins, which have different Mw between the experimental and the predicted ones. The proteins in the area enclosed by the dotted lines denote that they have an experimental Mw within ± 25% of the predicted molecular mass.

We estimated the molecular weight of the protein spots on the 2-DE gels and compared them with the theoretical molecular weight of strain SH100. While most of the estimated molecular weight values matched the theoretical values, we found 14 protein spots on the map that had different experimental and predicted molecular weights values (Figure 2). These proteins might be post-translationally modified by proteolytic processing, phosporylatoin of multiple amino acid residues and/or an artifact caused by sample preparation. For example, the experimental molecular weight of OmpA indicated that the protein was likely processed by a proteolytic enzyme, because two different spots (spot nos. 152 and 287) were identified as OmpA, the experimental masses of which were significantly lower than the theoretical values. Similar results were described in other reports [28,29].

Next, we classified proteins identified on the map using the KEGG pathway database. While 156 proteins (45.3%) were classified into several metabolic categories (carbohydrate, energy, lipid, nucleotide, amino acid, and other amino acids), 70 proteins (22.8%) were grouped in the no entry category, which means that these proteins do not belong to the other categories. This category contained 20 known virulence-associated proteins, including flagella and flagella biosynthesis proteins (FliC, FljB, FliY, FliG, FliM, and FliD), SPI-1 effectors (SipD, SopB, and SopE2), an SPI-1 translocase (SipC), an iron transporter (SitA), superoxide dismutases (SodA, SodB, SodC1, and SodC2), a quorum-sensing protein (LuxS), a two-component response regulator (PhoP), peptidyl-prolyl *cis-trans *isomerases (FkpA and SurA), and a periplasmic disulfide isomerase (DsbA).

### Identification of ppGpp-regulated proteins using comparative proteomics

**Figure 3:**
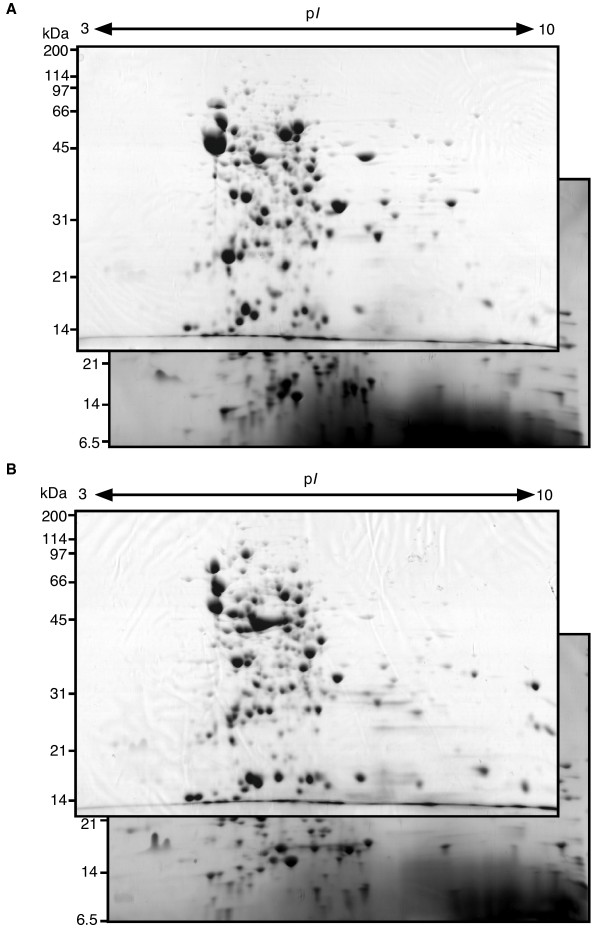
**Comparison of the agarose 2-DE maps of *S*. Typhimurium wild-type SH100 (A) and ppGpp-deficient strain TM157 (B) during amino acid starvation**. Both strains were grown under the same condition as described in Figure 1. Gels were stained with Coomassie Brilliant Blue.

To identify proteins associated with the stringent response in *S*. Typhimurium, we compared the agarose 2-DE pattern for each total protein prepared from amino acid-starved *S*. Typhimurium SH100 and Δ*relA*Δ*spoT *strain (TM157) (Figure 3). As shown in Table 1, 24 protein spots (23 proteins) were found at higher levels in SH100 than in TM157, while 23 protein spots were found at lower levels in SH100 than in TM157. We focused on 23 proteins, which were positively regulated by ppGpp in the stringent response.

**Table 1:** S. Typhimurium proteins regulated by ppGpp

**spot no**.	**STM no**.	Gene	Fold	Anova (*p*)	Average fold change determined by qRT-PCR
**Proteins expressed lower in Δ*relA*Δ*spoT *strain**					
002, 091	STM2884	*sipC*	0.1	0.006	ND^a^
005	STM0781	*modA*	0.3	0.032	0.67 ± 0.22
012	STM3169	*Stm3169*	0.3	0.004	0.18 ± 0.01^c^
014, 213	STM1796	*treA*	0.7	0.002	EC^b^
015	STM4403	*cpdB*	0.6	0.011	0.25 ± 0.06^c^
027	STM1954	*fliY*	0.5	0.033	ND
028	STM2884	*sipC*	0.1	0.009	ND
029	STM3557	*ugpB*	0.4	0.019	EC
029-2	STM0748	*tolB*	0.4	0.019	0.25 ± 0.03^c^
037	STM0209	*htrA*	0.6	0.032	0.60 ± 0.35
040	STM2638	*rseB*	0.3	0.011	0.88 ± 0.35
040-2	STM1478	*ydgH*	0.3	0.011	0.17 ± 0.06^c^
041	STM1375	*ynhG*	0.3	0.011	EC
056	STM1746	*oppA*	0.6	0.001	0.15 ± 0.05^c^
058	STM1746	*oppA*	0.5	0.006	0.15 ± 0.05^c^
059	STM1849	*yliB*	0.4	0.027	EC
060	STM3557	*ugpB*	0.3	0.006	EC
062	STM1091	*sopB*	0.2	0.036	ND
064	STM4319	*phoN*	0.1	0.014	0.54 ± 0.22
108	STM0435	*yajQ*	0.5	0.038	0.12 ± 0.05^c^
108-2	STM1440	*sodC1*	0.5	0.038	ND
153	STM3318	*yhbN*	0.6	0.047	0.28 ± 0.12^c^
154	STM4405	*ytfJ*	0.2	0.049	0.30 ± 0.02^c^
184	STM3348	*degQ*	0.4	0.038	EC
194	STM1720	*yciO*	0.3	0.028	14.22 ± 2.22^c^
**Proteins expressed higher in Δ*relA*Δ*spoT *strain**					
004	STM3359	*mdh*	2.0	0.021	ND
006	STM3069	*pgk*	1.4	0.037	ND
008	STM2681	*grpE*	1.5	0.018	ND
068	STM3342	*sspA*	1.7	0.014	EC
081	STM2952	*eno*	1.7	0.014	ND
096	STM1700	*fabI*	1.8	0.041	ND
098	STM0232	*accA*	2.2	0.017	ND
101	STM3446	*fusA*	3.7	0.022	ND
109	STM4055	*sodA*	2.0	0.044	EC
115	STM3415	*rpoA*	1.5	0.043	EC
116	STM4184	*aceA*	1.6	0.007	ND
118	STM0737	*sucB*	1.7	0.006	ND
119	STM2660	*clpB*	3.7	0.035	ND
135	STM0735	*sdhB*	2.1	0.002	ND
142	STM3063	*rpiA*	1.8	0.022	ND
145	STM4190	*pepE*	1.5	0.003	ND
155	STM0734	*sdhA*	2.9	0.039	ND
186	STM3282	*pnp*	3.2	0.013	ND
187	STM3446	*fusA*	2.3	0.031	ND
210	STM1305	*astD*	1.8	0.007	EC
222	STM3502	*ompR*	1.7	0.025	ND
227	STM2378	*fabB*	1.6	0.035	ND
231	STM1746	*oppA*	1.8	0.012	ND

^a^ND, not determined.

^b^EC, already identified as a ppGpp-regulated protein in *E. coli *by Traxler *et al. *[30].

^c^mRNA level was significantly different between wild type and the Δ*relA*Δ*spoT *mutant.

Of these proteins, six genes (*treA*, *ugpB*, *ynhG*, *yliB*, *ugpB*, *degQ*) had previously been identified as ppGpp-regulated genes in *E. coli *at the transcriptional level [30]. In *S*. Typhimurium, it has been shown that ppGpp controls the expression of known virulence-associated genes, including *sipC*, *fliY*, *sopB*, and *sodC1*, in response to growth conditions relevant to host infection [14]. Thus, to confirm the results from the comparative proteomic analysis, mRNA levels of the remaining 13 genes were assessed by qRT-PCR. As a result, mRNA expression levels of eight genes (*stm3169*, *cpdB*, *tolB*, *ydgH*, *oppA*, *yajQ*, *yhbN*, *ytfJ*) were significantly higher in SH100 than in TM157 under stringent conditions (Table 1).

### Identification of novel virulence-associated factors regulated by ppGpp

**Figure 4:**
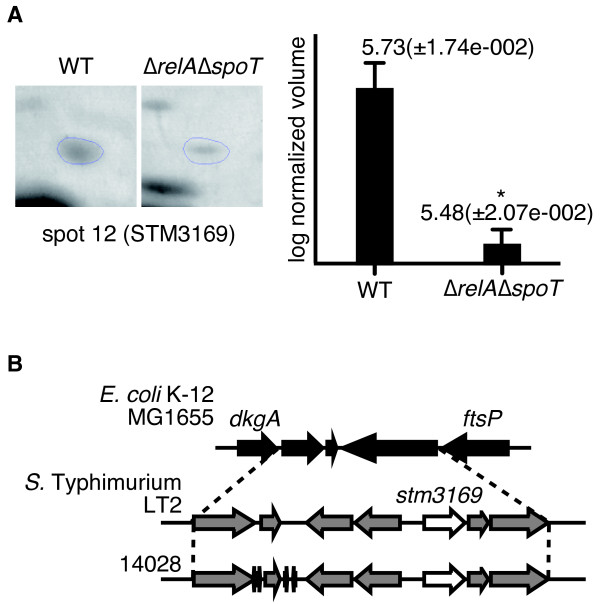
**The *S*. Typhimurium-specific protein STM3169 is regulated by ppGpp in the stringent response**. (A) Comparison of the STM3169 protein expression in the wild-type SH100 and Δ*relA*Δ*spoT *strain (TM157). An asterisk indicates that the difference was statistically significant (P < 0.05). (B) Genetic map of genes (open arrows) coding STM3169 within *Salmonella*-specific locus (gray arrows) and genes flanking the locus (closed arrows).

**Figure 5:**
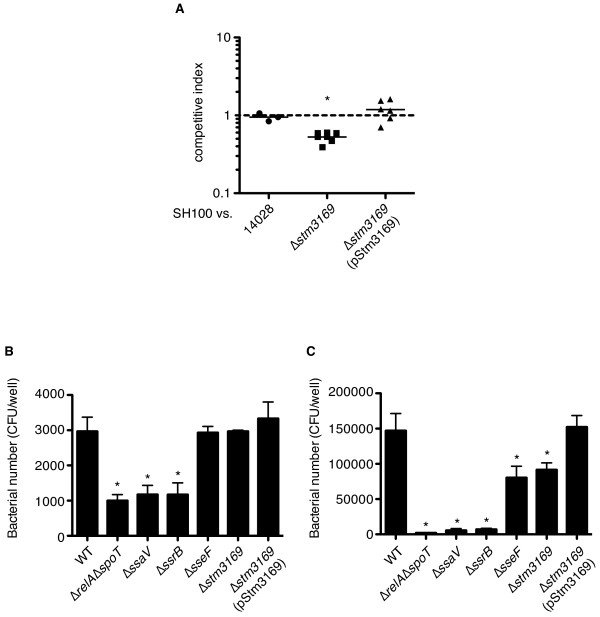
**STM3169 is a novel virulence protein**. Competitive index was determined at 48 h after infection in the spleen (A). Effects of *stm3169 *disruption on the invasion (B) and the intracellular survival (C) in the mouse macrophage cell lines, RAW264.7. Cells treated with IFN-γ were infected with *S*. Typhimurium wild-type and the mutant strains at a multiplicity of infection of 1. At 2 h and 24 h after infection, macrophages were lysed and the bacterial number was determined. Asterisks indicate that differences were statistically significant (*P* < 0.05).

Among 13 genes newly identified as ppGpp regulated, 12 genes were present in non-pathogenic *E. coli *K-12 strain. Therefore, to examine whether ppGpp-regulated putative or hypothetical proteins could contribute to the virulence of *S*. Typhimurium, we chose *Salmonella*-specific protein, STM3169, which is present in *S*. Typhimurium, but is absent in the *E. coli *K-12 strain (Figure 4[27,31]). To determine the roles of STM3169 in virulence, a deletion mutant was constructed in the *S*. Typhimurium wild-type SH100 strain, and its virulence was assessed by a mouse mixed infection using a competitive index analysis. As shown in Figure 5A, mouse mixed infections showed that disruption of the *stm3169 *gene conferred a defect in virulence in mice, and that successful complementation was achieved for TH973 (Δ*stm3169*::*kan*) by expression of intact STM3169 from a plasmid. These findings provide the first evidence that STM3169 functions as a virulence factor of *S*. Typhimurium in a mouse infection model.

Because it is believed that intracellular *Salmonella *is likely to be restricted to the acquisition of nutrient substrates from infected host cells, the stringent response could occur in SCV. Thus, we next analyzed the contribution of STM3169 to intracellular survival of *S*. Typhimurium in macrophages. In accordance with previous data that a ppGpp^0 ^mutant strain deficient in both *spoT *and *relA *genes resulted in a severe reduction of intracellular proliferation and suvival [12]. In contrast to the wild-type level of invasion, intracellular survival of TH973 in RAW264.7 cells was reduced, compared with that of the wild-type strain. The reduced CFU of TH937 in IFN-γ treated-RAW264.7 cells was not more severe than that in the Δ*relA*Δ*spoT *double mutant, Δ*ssaV *(SH113, SPI-2 T3SS component-defected mutant), and Δ*ssrB *(YY1, SPI-2 regulator mutant) strain, but was equal to that in the Δ*sseF *(TM548, SPI-2 effector mutant) strain (Figure 5B and 5C). These results suggest that the expression of additional virulence factors, like STM3169, in macrophages might be affected in a highly avirulent phenotype of a ppGpp-deficient strain in mice.

### *stm3169 *is regulated by the SPI-2 transcriptional regulator *ssrB*

**Figure 6:**
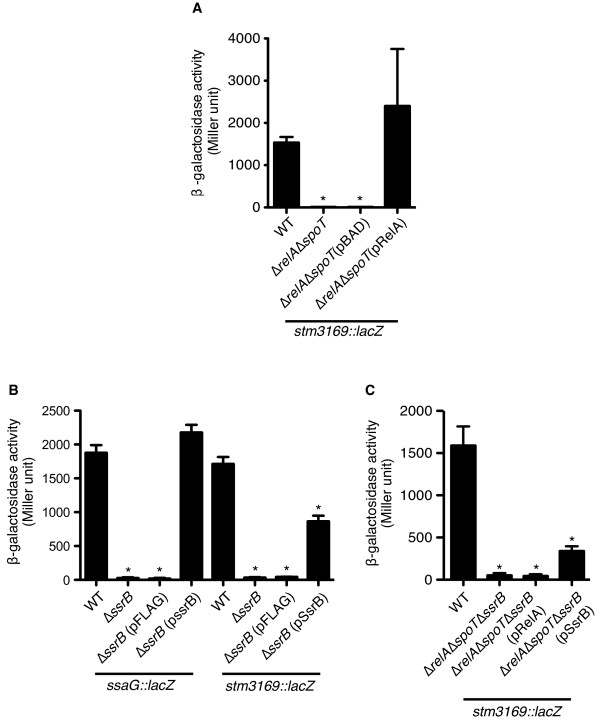
**STM3169 is regulated by ppGpp and *ssrB***. Transcriptional activity of *stm3169 *in *Salmonella *muntant strains. *Salmonella* Δ*relA*Δ*spoT *(A), Δ*ssrB* (B), and Δ*relA*Δ*spoT*Δ*ssrB* (C) mutant strains carrying *stm3196::lacZ *fusion were incubated in MgM medium (pH5.8) for 18 h. The promoter activity of *stm3169 *was estimated by mesuring the β-garactosidase activity. L-arabinose (a final concentration of 0.001%) and IPTG (a final concentration of 0.01 mM) were added in the medium for induction of RelA on pRelA and for SsrB on pSsrB, respectively. Asterisks indicate that differences were statistically significant (*P* < 0.05).

It has been demonstrated that ppGpp mediates the expression of virulence-associated genes involved in bacterial invasion and intracellular growth and survival via global and/or gene-specific transcriptional regulators in *S*. Typhimurium [12,14]. Since intracellular growth and suvival of *Salmonella *in macrophages is dependent upon SPI-2 function, we next confirmed whether expression of *stm3169 *is regulated by the SsrAB two-component system, which positively controls the expression of SPI-2 genes as well as other genes belonging to the SsrB regulon [32]. To test this, we constructed *S*. Typhimurium strains carrying *stm3169*::*lacZ *transcriptional fusions on the chromosome in the wild-type (SH100) and Δ*relA*Δ*spoT *(TM157) genetic background. *Salmonella *strains carrying the *stm3169*::*lacZ *fusion gene (TH1162 and TH1164) were grown in defined MgM medium (pH 5.8) with 0.1% casamino acids and measured β-galactosidase activity. The transcription levels of *stm3169*::*lacZ *fusion were significantly decreased in TM157 (Figure 6A). The reduced level was restored to the wild-type level by the introduction of an arabinose-inducible plasmid expressing His_6_-tagged RelA protein. We next transduced the *ssrB *mutation (Δ*ssrB*::*cat*) into a *stm3169*::*lacZ *fusion strain (TH1162). Strains carrying the *stm3169*::*lacZ *fusion gene with the *ssrB *mutation were grown in MgM medium (pH 5.8), and β-galactosidase activity was measured. Control experiments were performed with the *ssaG*::*lacZ *fusion gene (TM129). *ssaG *expression is strongly controlled by SsrB [33]. Similar to *ssaG*::*lacZ*, the transcription level of the *stm3169*::*lacZ *fusion gene was significantly decreased in strains carrying the *ssrB *mutation (Figure 6B). Complementation was partially achieved for TM423 by expression of SsrB (SsrB-FLAG) on a plasmid (Figure 6B), probably due to the constitutive expression of SsrB from multi-copy-number palsmid pFLAG-CTC. Collectively, these data suggest that the novel virulence-associated factor STM3169 was regulated by the SPI-2 two-component regulatory system SsrAB as well as by ppGpp.

It has been reported that ppGpp regulates SPI-2-encoded genes under aerobic condition [14]. To further characterize the transcriptional regulation of *stm3169 *by ppGpp and SsrB, we constructed a Δ*relA*Δ*spoT*Δ*ssrB *triple mutant strain (YY2), and examined the affect of the transcriptional activity on *stm3169*::*lacZ *fusion gene. While the transcriptional activity of *stm3169*::*lacZ *fusion in the triple mutant strain was significantly reduced at the same level of Δ*relA*Δ*spoT *double mutant strain, it could be restored by introduction of plasmid pSsrB expressing SsrB-FLAG but not pRelA expressing His_6_-tagged RelA (Figure 6C). These results indicate that ppGpp is controlled the expression of *stm3169 *through SsrB.

STM3169 is homologous to DctP in *Rhodobacter capsulatus *with a 31% identity and a 73% similarity. DctP, along with DctQ and DctM, constitutes a tripartite ATP-independent periplasmic transporter (TRAP-T) system involved in succinate utilization, and DctP plays a role as an extracytoplasmic solute receptor in this transporter [34]. STM3170 and STM3171, which are located immediately downstream from STM3169, have a 66% and an 80% similarity with DctQ and DctM, respectively. These suggest that the TRAP-T in *S*. Typhimurium is composed of *stm3169*, *stm3170*, and *stm3171 *genes. In addition, two hypothetical operons, *yiaOMN *and *stm4052-4054*, are annotated as TRAP-T in the *S*. Typhimurium strain LT2 [31]. Recently, it has been reported that the TRAP-T (SiaPQM) in *Haemophilus influenzae *is essential for LPS sialylation and virulence [35]. Further research is necessary to determine the role of these transporters in *S*. Typhimurium virulence.

## Conclusions

We constructed an agarose 2-DE reference map of amino-acid starved *S*. Typhimurium and identified a novel virulence-associated factor, STM3169, regulated by ppGpp by applying the map to comparative proteomics. *stm3169 *is also regulated by an SPI-2 two-component regulator, SsrB. Recently, it has been reported that the lack of ppGpp synthesis in *Salmonella *strains attenuates virulence and induces immune responses in mice [36]. Thus, further analysis of proteins regulated by ppGpp may lead to the development of new vaccines.

## Methods

### Bacterial strains, primers, and culture conditions

The bacterial strains and plasmids used in this study are listed in Table 2. The oligonucleotide primers used are listed in Table 3. Bacteria were grown in Luria-Bertani (LB) medium or on LB agar under conditions suitable for selection for resistance to ampicillin (100 μg/mL), chloramphenicol (25 μg/mL), nalidixic acid (50 μg/mL), or spectinomycin (50 μg/mL), as appropriate. To induce the bacterial stringent response, serine hydroxamate (Sigma; 0.005%), an inhibitor of serine tRNA synthetase, was added to a 12 h culture in LB broth, and the bacteria were further incubated for 1 h [26]. Magnesium minimal medium (MgM, pH 5.8) was used to induce SPI-2 gene expression [6].

**Table 2:** Bacterial strains and plasmids used.

Strains	Relevant characteristics	**Source/Ref**.
**Bacterial strains** *S*. Typhimurium		
14028	wild-type	ATCC
SH100	Spontaneous nalidixic acid resistant derivative of wild-type 14028	[44]
TM157	SH100 Δ*relA*::*cat *Δ*spoT*::*kan*	this study
YY2	SH100 Δ*relA*::*cat *Δ*spoT*::*kan *Δ*ssrB*::*tet*	this study
TH973	SH100 Δ*stm3169*::*kan*	this study
TH1162	SH100 *stm3169*::*lacZ*	this study
TH1164	TM157 *stm3169*::*lacZ*	this study
YY3	TH1164 Δ*ssrB*::*tet*	this study
TM129	SH100 *ssaG*::*lacZ*	this study
YY1	SH100 Δ*ssrB*::*tet*	this study
SH113	SH100 Δ*ssaV*::*cat*	[11]
TM548	SH100 Δ*sseF*::*kan*	this study
*E. coli*		
DH5α	K-12 *recA1 endA1 gyrA96 thi-1 hsdR17 supE44 *(*lacXYA-argR*)*U169 deoR *(*80 dlac *(*lacZ*)*M15*)	Invirtogen
SM10λ*pir*	*thi-1 thr leu tonA lacY supE recA*::RP4-2-Tc::Mu λ*pir*	[45]
**Plasmids**		
pGEM-Teasy	TA cloning vector	Promega
pMW118	pSC101-based low copy number plasmid	Nippon Gene
pACYC184	p15A-based low copy number plasmid, *tet *template	New England Biolabs
pFLAG-CTC	FLAG tag expression vector	Sigma
pLD-*lacZ*Ω	Integrational plasmid with promoterless *lacZ *gene	[39]
pBAD-HisA	Expression vector for His6 fusion protein	Invitrogen
pMW-Stm3169	*stm3169 *gene in pMW118	this study
pLD-stm3169Z	*stm3169*::*lacZ *operon fusion in pLD-*lacZ*Ω	this study
pLD-ssaGZ	*ssaG*::*lacZ *operon fusion in pLD-*lacZ*Ω	this study
pRelA	pBAD-HisA expressing *relA *gene	this study
pSsrB	pFLAG-CTC expressing *ssrB *gene	this study
pKD46	Red recombinase expression plasmid	[41]
pKD4	*kan *cassette used for gene deletion	[41]

**Table 3:** Primers

Name	Nucleotide sequence (5' to 3')*^a^*
**Construction of the deletion mutants**	
relA-FW	CGCCATCCCGCAATGGTTTACATAA
relA-RV	TCATTGTTCTGGCCATAACAGC
spoT-FW	CTTGAAAACCATCATTCGCGCTGAACG
spoT-RV	TCTGCGGTACGAATGATTGCAGAAACG
stm3169-red-FW	ACGTTCATTCACAACATCAGCGGTATTACTGGCCGGCTGTGTGTAGGCTGGAGCTGCTTC
stm3169-red-RW	ACATATTCTCGATGTATTCCAGATCCTTCGCTGACTGAGCCATATGAATATCCTCCTTAG
sseF-red-FW	AACAGAACGAAATATGAAAATTCATATTCCGTCAGCGGCAGTGTAGGCTGGAGCTGCTTC
sseF-red-RW	TGTCCATTAATGCAGGTGTAGTAGCAGATTGACAGAGCGCCATATGAATATCCTCCTTAG
pAC-tet-FW	TTGGTAGCTCAGAGAACCTTCGAAAAACCG
pAC-tet-RV	TCGCTCGCGTATCGGTGATTCATTCGCTA
**Construction of plasmids for the complementations**	
relA-FW2	AGGCTCGAGGTCGCGGTAAGAAGT
relA-RV2	ACAAGCTTACTGTCTGGGGTTTAC
ssrB-FW	GGGCTCGAGGAATATAAGATCTTATTAGTA
ssrB-RV	CCCGGATCCATACTCTATTAACCTCATTCT
stm3169-FW	CCGCTCGAGAACACACGTTCATTCACAACATCAG
stm3169-RV	GGAAGATCTATTCTCGATGTATTCCAGATCCTTC
**Construcion of the *lacZ *fusions**	
ssaG-Pro-FW	AAAGTCGACCAAATGCTCAGGTAGGAGGGC
ssaG-Pro-RV	AAAGGATCCATCATCGATTCTGGGTTGAGC
stm3169-Pro-FW	ACGCGTCGACGACGATTTAGCCGGTATGAAAATCA
stm3169-Pro-RV	CGGGATCCTTACATATTCTCGATGTATTCCAGA
**Comfirmation of gene expression by qRT-PCR**	
gyrA-FW	AAGAGCTCCTATCTGGATTATGC
gyrA-RV	TATTTACCGATTACGTCACCAAC
relA-FW	ATTGTGCCATTCACCTATCAGTT
relA-RV	GATATTTTTGTCACGATCCTGCT
invF-FW	ATCGCTGCTGAATAGTGTAGAAG
invF-RV	CATTTGTCTGCCAATTGAATAAT
stm0209-FW	CCTGAACGTAGAAAATTACGAGA
stm0209-RV	GTCAGGTTTTTCACCATGTTACT
stm0435-FW	GTCAATCAGTTGCTCGATATTCTG
stm0435-RV	TTTAATCAGCTTGACGATTTTCTTC
stm0748-FW	TGAACCTGTACGTTATGGATCTC
stm0748-RV	CGCCGTTAATGTTCATTTTATAC
stm0781-FW	GAAGGCAAGATCACCGTATTT
stm0781-RV	CTGATCAGCAGAGATGAAGAGAT
stm1478-FW	ACAAAAGTTGAGGAGCTGAATAAAG
stm1478-RV	GCCACTGACGCGTAATATGATAA
stm1720-FW	TTGGTTGTAAAATTGAAGACAAAGG
stm1720-RV	GTCCCCTTCAGGATAAAGGTGTAAT
stm1746-FW	CGAATTATTCCAGAAACTGAAGAAA
stm1746-RV	ATCGCCCTGATTTTTAACCTTATTA
stm2638-FW	TATTCTGACGGTCTGTTTAGCTTTT
stm2638-RV	GTACTGCCCTGAATTTGATACTGTC
stm3169-FW	GTTACCAGAATAATGTCGCAGCTAT
stm3169-RV	AATCATCCACATAAAAAGAATCTGG
stm3318-FW	CAAACTCAGCCTTAATCTTATGC
stm3318-RV	ACTTTATCGGCGTTGATCTTAAT
stm4319-FW	ATTAGTATTATCCGAGGCCAGAC
stm4319-RV	CAGTCTTGCAAACTCTACTGCTC
stm4403-FW	ATTGATATTCACAGCAACAAACC
stm4403-RV	AGGTCAGGTTTTTAATACGTTCC
stm4405-FW	CGAACTGACGTTGAATAAAGATGAG
stm4405-RV	AATTGTGGTCGTCTGGTATCTGT

^a^Underlined part indicates P1 or P2 site for pKD4, or restriction sites.

### Construction of mutants

Nonpolar mutants of *relA *and *spoT *were constructed by allele exchange using the temperature- and sucrose-sensitive suicide vector pCACTUS [37]. The *relA *and *spoT *genes were amplified by PCR with the following primers: (1) relA-FW and relA-RV for *relA *and (2) spoT-FW and spoT-RV for *spoT. S*. Typhimurium strain SH100 genomic DNA was used as the template. The PCR products were cloned into TA cloning vector pGEM-T Easy (Promega) generating plasmid pGEM-*relA *and pGEM-*spoT*, respectively. A disruption mutation of *relA *was created by the insertion of the HincII-digested promoterless *cat *gene into a unique NruI site in the coding sequence of *relA *on pGEM-*relA*. The resulting plasmid pGEM-*relA*::*cat *was digested with BglII and then self-ligated, yielding plasmid pGEM-Δ*relA*::*cat*. In contrast, the *spoT *gene was disrupted by the insertion of a SmaI-digested Km^r^-encoding gene (*kan*) cassette from pUC18K [38] into NruI sites in the coding sequence of *spoT *on pGEM-*spoT*, thus generating pGEM-Δ*spoT*::*kan*. The disrupted gene was then subcloned using SalI and SphI into similarly digested pCACTUS, and the resulting plasmid was introduced into strain SH100 by electroporation for allele exchange mutagenesis, which was carried out as described previously [39]. Δ*relA*Δ*spoT *mutant strain was created by phage P22-mediated transduction [40].

The PCR-based λ Red recombinase system using pKD46 and pKD4 was performed to disrupt *stm3169 *or *sseF *[41]. The growth rate of these mutant strains in LB and MgM (pH5.8) broth showed the same levels to wild-type strain.

To construct Δ*relA*Δ*spoT*Δ*ssrB *mutant strain, the cloned *ssrB *gene was disrupted by the insertion of a Tet^r^-encoding gene (*tet*) cassette, which was amplified with pAC-tet-FW and pAC-tet-RV primers using pACYC184 (New England Biolabs) as template. The Δ*ssrB*::*tet *fragment was amplified by PCR using ssrB-FW and ssrB-RV primers, and the resulting PCR product was introduced into *S*. Typhimurium SH100 carrying pKD46. The disrupted genes were transferred by phage P22 transduction into Δ*relA*Δ*spoT *mutant strain TM157.

To construct *ssaG*::*lacZ *and *stm3169*::*lacZ *transcriptional fusions, pLD-ssaGZ and pLD-stm3169Z were transferred from *Escherichia coli *SM10λ*pir *to *S*. Typhimurium SH100 by conjugation. The fusions were introduced into SH100, Δ*relA*Δ*spoT *(TM157), Δ*ssrB*::*tet *(YY3), and Δ*ssaV *(SH113) mutant strains by phage P22-mediated transduction. All constructs were verified by PCR or DNA sequencing.

### Construction of plasmids

For construction of the complementing plasmid, pMW-Stm3169, *stm3169 *gene was amplified by PCR with stm3169-FW and stm3169-RV primers. *S*. Typhimurium SH100 genomic DNA was used as the template. The PCR products were digested with BglII and XhoI, and cloned into the Bglll-XhoI site on pMW118 (Nippon Gene), generating plasmid pMW-Stm3169.

To construct pRelA and pSsrB, the target genes were amplified by PCR with the following primers: relA-FW2 and relA-RV2 for *relA *and ssrB-FW and ssrB-RV for *ssrB*. The PCR product containing *relA *was digested with XhoI-HindIII and cloned into the same sites on pBAD-HisA (Invitrogen). The PCR product containing *ssrB *was digested with XhoI-BamHI and cloned into the same sites on pFLAG-CTC (Sigma). pRelA and pSsrB expressed His_6_-tagged RelA and SsrB-FLAG fusion protein, respectively.

To construct *lacZ *transcriptional fusions, the DNA fragments containing (predicted) promoter regions of *ssaG *were amplified by PCR using the primers ssaG-Pro-FW and ssaG-Pro-RV, and those containing promoter regions of *stm3169 *were amplified using stm3169-Pro-FW and stm3169-Pro-RV. The PCR products digested with SalI and BamHI were ligated into the same sites of pLD-*lacZ*Ω [39].

### Sample preparation for agarose 2-DE

Agarose 2-DE samples were prepared from amino-acid starved *S*. Typhimurium strain SH100, as well as *relA *and *spoT *double knockout strain TM157 (Δ*relA*Δ*spoT*). The cell pellets were washed twice with cold phosphate-buffered saline (PBS) and dissolved in lysis buffer containing 5 M urea, 1 M thiourea, 0.05% w/v β-mercaptoethanol, and one tablet of protein inhibitor (Complete Mini EDTA-free; Roche Diagnostics, Mannheim, Germany), which was dissolved in 10 mL of the solution. The lysates were centrifuged (104,000 × *g*, 20 min, 4°C) and the clear supernatant was used.

### Proteome analysis

We performed proteome analysis according to the procedures of Oh-Ishi *et al. *[25] and Kuruma *et al. *[42]. An aliquot of 200-300 μL (containing 500 μg of protein) of sample solution was subjected to first-dimension IEF at 667 V for 18 h at 4°C, followed by second-dimension SDS-PAGE. The slab gel was stained with CBB R-350 (PhastGel Blue R; GE Healthcare).

Protein spots were excised from a destained gel with 50% (v/v) ACN and dried under vacuum. The proteins were digested in the gel with trypsin. Digested fragments of 15 pmol were loaded on a Liquid Chromatography-Mass Spectrometry/Mass Spectrometry (LC-MS/MS), which consisted of Nanospace SI-2 (Shiseido Fine Chemicals), an HPLC (LCQ Deca), and an ion trap mass spectrometer (Thermo Finnigan). We identified a protein from measured masses of the tryptic peptides and their MS/MS fragments using the SEQUEST program. When the top-ranked candidates had SEQUEST scores lower than 90, we inspected the raw MS and MS/MS spectra of peptides to judge their qualities. We classified identified proteins according to the Kyoto Encyclopedia of Genes and Genomes (KEGG) PATHWAY database http://www.genome.ad.jp/kegg/pathway.html.

Gel-to-gel comparisons between SH100 and TM153 were performed for two separately prepared samples. Each scanned 2-DE gel image was analyzed with the gel image analysis software SameSpots (Progenesis).

### RNA extraction and quantitative real-time PCR

*S*. Typhimurium strains were grown in LB and ppGpp expression was induced as described above. Total RNA was isolated from the bacterial culture using RNAprotect Bacteria Reagent and the RNeasy Protect Bacteria Mini Kit with the gDNA Eliminator spin column (Qiagen) according to the manufacturer's instructions. cDNA was synthesized using the QuantiTect Reverse Transcription Kit (Qiagen). Real-time PCR was performed with the primer pairs listed in Table 3 using QuantiTect SYBR Green and the 7900HT Sequence Detection System (Applied Biosystems). The data were analyzed using the comparative Ct method (Applied Biosystems). Transcription of the target gene was normalized to the levels of *gyrA *mRNA.

### Mouse infections

For the competitive index assay, female BALB/c mice (5-6 weeks old) were used for the mouse infection study and were housed at Kitasato University according to the standard Laboratory Animal Care Advisory Committee guidelines. Mice were inoculated by intraperitoneal infection with 100 μL of inoculum containing a total of 1 × 10^5 ^bacteria (each strain at 5 × 10^4^), consisting of an equal number of wild-type and mutant strains. At 48 h after infection, the mice were sacrificed by carbon dioxide inhalation. The spleens were homogenized in cold PBS by mechanical disruption. The number of each strain in the spleen was determined by plating a dilution series of the lysate onto LB agar alone and LB agar with appropriate antibiotics. Each competitive index value was calculated as [mutant/wild-type] output/[mutant/wild-type] input and represented as the mean of at least three independent infections.

### Macrophage survival assay

Cells of a mouse macrophage-like line, RAW264.7, were diluted in DMEM containing 10% FBS and seeded in 24-well plates at a density of 5 × 10^5 ^cells per well. *S*. Typhimurium strains were used to infect RAW264.7 cells at a multiplicity of infection of 1. The bacteria were centrifuged onto the cells (500 ×*g*, 5 min) and incubated for 25 min at 37°C in a 5% CO_2 _incubator. Cells were washed three times with PBS, and DMEM containing interferon-γ (IFN-γ) (100 units/well; Peprotech) and gentamicin (100 μg/mL; Sigma) was added. After 95 min of incubation, the medium was replaced with DMEM containing IFN-γ (100 units/well) and gentamicin (10 μg/mL). The number of intracellular bacteria was determined at 2 h and 24 h after infection. For the enumeration of intracellular bacteria, the cells were washed three times with PBS and lysed in 1% Triton X-100, and bacteria were quantified by spreading serial 10-fold dilutions of RAW264.7 cell lysates on LB agar plates to count the colony-forming units (CFU). Each experiment was repeated three times.

### β-galactosidase assay

β-galactosidase activities of reporter gene fusions were determined according to a standard procedure [43].

### Statistical analysis

The competitive index, mRNA expression, and bacterial proliferation in macrophage cells were compared using Student's t-test. For comparative proteomics, the intensity of the spot was compared by one-way ANOVA. Values of *P *< 0.05 were considered statistically significant.

## Authors' contributions

TH, SM, YYO, YKo, and SSI performed the experiments. TH and NO designed the experiments. TMi constructed the TM157, TM129, and TM548 strains. YKu assisted with the experiments. MOI, TMa, and HD advised regarding the design of the experiments. TH and NO wrote the paper.

## Supplementary Material

Additional file 1 **Table S1**. Proteins identified on the reference map. Click here for file
