# Supplementary material for: Comparative proteomic analysis of Salmonella enterica serovar Typhimurium ppGpp-deficient mutant to identify a novel virulence protein required for intracellular survival in macrophages
Source: BMC Microbiol. 2010 Dec 21;10:324. doi: 10.1186/1471-2180-10-324 (PMC3022708; doi:10.1186/1471-2180-10-324)
Supplement: Additional file 1 — Table S1. Proteins identified on the reference map. [file 1471-2180-10-324-S1.PDF]

**Table S1. Proteins identified on the reference map**

| Spot no. | STM no. | Gene type | Alternative gene symbol | Mw[a] | Score[b] | Seq. cov. (%)[c] |
|---|---|---|---|---|---|---|
| 1 | STM1959 | *fliC* | flagellar biosynthesis; flagellin, filament structural protein | 51593 | 140.4 | 31.3 |
| 1-2 | STM2771 | *fljB* | Flagellar synthesis: phase 2 flagellin (filament structural protein) | 52517 | 90.0 | 21.7 |
| 2 | STM2884 | *sipC* | cell invasion protein | 42965 | 204.3 | 45.2 |
| 3 | STM1290 | *gapA* | glyceraldehyde-3-phosphate dehydrogenase A | 35568 | 295.8 | 53.2 |
| 4 | STM3359 | *mdh* | malate dehydrogenase | 32457 | 213.2 | 44.9 |
| 5 | STM0781 | *modA* | ABC superfamily (peri_perm), molybdate transporter | 27502 | 226.0 | 61.1 |
| 6 | STM3069 | *pgk* | phosphoglycerate kinase | 41114 | 237.4 | 57.1 |
| 7 | STM0217 | *tsf* | protein chain elongation factor EF-Ts | 30339 | 86.1 | 47.3 |
| 8 | STM2681 | *grpE* | molecular chaparone; heat shock protein | 21882 | 103.7 | 32.1 |
| 9 | STM2433 | *crr* | PTS family, glucose-specific IIA component | 18229 | 99.1 | 42.6 |
| 10 | STM4055 | *sodA* | superoxide dismutase, manganese | 23061 | 109.2 | 39.8 |
| 11 | STM3453 | *fkpA* | FKBP-type peptidyl-prolyl cis-trans isomerase (rotamase) | 28926 | 178.0 | 40.8 |
| 12 | STM3169 | *stm3169* | putative dicarboxylate-binding periplasmic protein | 36198 | 94.5 | 26.9 |
| 13 | STM0890 | *artI* | ABC superfamily (bind_prot), arginine transport system | 26978 | 114.1 | 32.9 |
| 14 | STM1796 | *treA* | trehalase, periplasmic | 63491 | 220.1 | 32.5 |
| 15 | STM4403 | *cpdB* | 2':3'-cyclic-nucleotide 2'-phosphodiesterase | 70498 | 201.9 | 30.1 |
| 16 | STM2166 | *bglX* | beta-D-glucoside glucohydrolase, periplasmic | 83374 | 290.7 | 33.2 |
| 17 | STM0964 | *dmsA* | anaerobic dimethyl sulfoxide reductase, subunit A | 90328 | 194.0 | 21.3 |
| 18 | STM0092 | *surA* | peptidyl-prolyl cis-trans isomerase, survival protein | 47232 | 229.4 | 49.8 |
| 19 | STM3551 | *ggt* | gamma-glutamyltranspeptidase | 61702 | 228.1 | 31.0 |
| 20 | STM0007 | *talB* | transaldolase B | 35153 | 153.6 | 48.6 |
| 21 | STM3068 | *fba* | fructose-bisphosphate aldolase | 39138 | 129.9 | 30.4 |
| 22 | STM1210 | *ycfP* | putative esterase | 21059 | 56.1 | 28.9 |
| 23 | STM1431 | *sodB* | superoxide dismutase, iron | 21289 | 67.9 | 21.2 |
| 24 | STM1044 | *sodC* | Gifsy-2 prophage: superoxide dismutase precursor (Cu-Zn) | 18352 | 53.3 | 48.0 |
| 25 | STM0665 | *gltI* | ABC superfamily (bind_prot), glutamate/aspartate transporter | 34114 | 119.4 | 40.9 |
| 26 | STM3884 | *rbsB* | ABC superfamily (peri_perm), D-ribose transport protein | 30944 | 172.3 | 49.0 |
| 27 | STM1954 | *fliY* | putative periplasmic binding transport protein | 28787 | 230.4 | 61.7 |
| 28 | STM2884 | *sipC* | cell invasion protein | 42965 | 193.4 | 45.0 |
| 29 | STM3557 | *ugpB* | ABC superfamily (peri_perm), sn-glycerol 3-phosphate transport protein | 48374 | 130.3 | 27.2 |
| 29-2 | STM0748 | *tolB* | tol protein required for outer membrane integrity, uptake of group A colicins, and translocation of phage DNA to cytoplasm, may be part of multiprotein peptidoglycan recycling complex (Two domains) | 46130 | 105.4 | 28.4 |
| 30 | STM3310 | *yrbC* | putative ABC superfamily (atp&memb), transport protein | 23986 | 156.8 | 50.7 |
| 31 | STM2165 | *yehZ* | putative ABC superfamily (bind_prot) transport protein (possibly glycine betaine choline transport for osmoprotection) | 32739 | 149.2 | 24.9 |
| 32 | STM2354 | *hisJ* | ABC superfamily (bind_prot), histidine transport protein | 28361 | 127.4 | 37.7 |
| 33 | STM2355 | *argT* | ABC superfamily (bind_prot), lysine/arginine/ornithine transport protein | 28181 | 139.8 | 37.7 |
| 34 | STM0168 | *yacK* | putative multicopper oxidase | 58607 | 79.1 | 17.2 |
| 35 | STM1679 | *mppA* | periplasmic murein tripeptide transport protein, also negative regulator of mulitple antibiotic | 59859 | 257.5 | 43.9 |
| 36 | STM2494 | *stm2494* | putative inner membrane or exported | 53722 | 140.0 | 29.6 |
| 37 | STM0209 | *htrA* | periplasmic serine protease Do, heat shock protein | 49297 | 187.3 | 33.7 |
| 38 | STM1165 | *grxB* | glutaredoxin 2 | 24413 | 128.5 | 41.4 |
| 39 | STM4232 | *malM* | periplasmic protein of mal regulon | 31805 | 136.4 | 28.2 |
| 40 | STM2638 | *rseB* | anti sigma E (sigma 24) factor, negative regulator | 35749 | 128.9 | 31.8 |
| 40-2 | STM1478 | *ydgH* | putative periplasmic protein | 33897 | 75.2 | 27.1 |
| 41 | STM1375 | *ynhG* | putative LysM domain | 36111 | 98.6 | 30.3 |
| 42 | STM3857 | *pstS* | ABC superfamily (bind_prot), high-affinity phosphate transporter | 36798 | 168.6 | 48.3 |
| 43 | STM4582 | *slt* | lytic murein transglycosylase, soluble | 75066 | 213.0 | 30.3 |
| 44 | STM1091 | *sopB* | Salmonella outer protein: homologous to ipgD of Shigella | 61916 | 184.0 | 37.1 |
| 45 | STM0617 | *rna* | RNase I, cleaves phosphodiester bond between any two nucleotides | 29509 | 63.8 | 18.7 |
| 46 | STM4148 | *nusG* | component in transcription antitermination | 20527 | 158.1 | 57.5 |
| 47 | STM4570 | *deoD* | purine-nucleoside phosphorylase | 25959 | 102.2 | 24.7 |
| 48 | STM0837 | *ybiS* | putative periplasmic protein | 33274 | 160.9 | 42.8 |
| 49 | STM3355 | *stm3355* | putative tartrate dehydratase alpha subunit | 32224 | 64.5 | 24.4 |
| 50 | STM1891 | *znuA* | ABC superfamily (bind_prot) high affinity Zn transport protein | 36459 | 129.1 | 25.2 |
| 50-2 | STM4077 | *yneA* | putative ABC superfamily (peri_perm), sugar transport protein | 36741 | 55.4 | 23.2 |
| 51 | STM3106 | *ansB* | periplasmic L-asparaginase II | 36908 | 248.0 | 50.0 |
| 52 | STM2282 | *glpQ* | glycerophosphodiester phosphodiesterase, periplasmic | 40406 | 176.5 | 41.0 |
| 53 | STM3567 | *livJ* | ABC superfamily (bind_prot), branched-chain amino acid transporter, high-affinity | 38769 | 105.4 | 40.5 |
| 53-2 | STM0977 | *serC* | 3-phosphoserine aminotransferase / phosphohydroxythreonine transaminase | 39814 | 54.1 | 16.0 |
| 54 | STM4007 | *glnA* | glutamine synthetase | 51767 | 147.4 | 27.1 |
| 54-2 | STM3136 | *stm3136* | putative D-mannonate oxidoreductase | 53989 | 52.6 | 11.0 |
| 55 | STM0224 | *yaeT* | putative outer membrane antigen | 89508 | 192.0 | 26.9 |
| 56 | STM1746 | *oppA* | ABC superfamily (periplasm), oligopeptide transport protein with chaperone properties | 65517 | 254.5 | 28.2 |
| 57 | STM2090 | *rfbH* | LPS side chain defect: CDP-6deoxy-D-xylo-4-hexulose-3-dehydrase | 48085 | 168.6 | 30.2 |
| 58 | STM1746 | *oppA* | ABC superfamily (periplasm), oligopeptide transport protein with chaperone properties | 65517 | 196.1 | 34.7 |
| 59 | STM0849 | *yliB* | putative ABC transporter periplasmic binding protein | 56520 | 228.1 | 45.9 |
| 60 | STM3557 | *ugpB* | ABC superfamily (peri_perm), sn-glycerol 3-phosphate transport protein | 48374 | 248.3 | 42.0 |
| 61 | STM2861 | *sitA* | Salmonella iron transporter: fur regulated | 33704 | 225.0 | 45.6 |
| 62 | STM1091 | *sopB* | Salmonella outer protein: homologous to ipgD of Shigella | 61916 | 199.2 | 31.2 |
| 63 | STM0665 | *gltI* | ABC superfamily (bind_prot), glutamate/aspartate transporter | 34114 | 75.9 | 29.9 |
| 64 | STM4319 | *phoN* | non-specific acid phosphatase | 28364 | 71.6 | 20.0 |
| 65 | STM2324 | *nuoF* | NADH dehydrogenase I chain F | 49227 | 144.0 | 28.3 |
| 66 | STM1780 | *prsA* | phosphoribosylpyrophosphate synthetase | 34166 | 139.6 | 45.1 |
| 66-2 | STM2190 | *mglB* | ABC superfamily (peri_perm), galactose transport protein | 35795 | 73.1 | 30.1 |
| 67 | STM1815 | *minD* | cell division inhibitor, a membrane ATPase, activates MinC, directs division apparatus to middle of cell by oscillating from one half to other | 29481 | 149.9 | 50.0 |
| 68 | STM3342 | *sspA* | stringent starvation protein A, regulator of transcription | 24230 | 137.8 | 52.8 |
| 69 | STM3630 | *dppA* | ABC superfamily (peri_perm), dipeptide transport protein | 60201 | 128.4 | 27.5 |
| 69-2 | STM0609 | *ahpF* | alkyl hydroperoxide reductase, F52a subunit; detoxification of hydroperoxides | 55931 | 43.2 | 10.7 |
| 70 | STM1117 | *agp* | glucose-1-phosphatase | 45540 | 128.5 | 25.2 |
| 71 | STM3142 | *stm3142* | putative ferrichrome-binding periplasmic protein | 39616 | 169.7 | 44.3 |
| 72 | STM0209 | *htrA* | periplasmic serine protease Do, heat shock protein | 49297 | 169.5 | 27.2 |
| 73 | STM2771 | *fljB* | Flagellar synthesis: phase 2 flagellin (filament structural protein) | 52517 | 153.7 | 37.4 |
| 73-2 | STM1959 | *fliC* | flagellar biosynthesis; flagellin, filament structural protein | 51593 | 107.3 | 26.7 |
| 74 | STM0012 | *dnaK* | chaperone Hsp70 in DNA biosynthesis/cell division | 69240 | 221.2 | 34.0 |
| 75 | STM0772 | *gpmA* | phosphoglyceromutase 1 | 28475 | 161.1 | 40.8 |
| 76 | STM2190 | *mglB* | ABC superfamily (peri_perm), galactose transport protein | 35795 | 173.4 | 40.4 |
| 77 | STM3630 | *dppA* | ABC superfamily (peri_perm), dipeptide transport protein | 60201 | 199.8 | 36.8 |
| 77-2 | STM3867 | *atpA* | membrane-bound ATP synthase, F1 sector, alpha-subunit | 55094 | 114.5 | 20.7 |
| 78 | STM4229 | *malE* | ABC superfamily (bind_prot) maltose transport protein, substrate recognition for transport and | 43465 | 209.5 | 54.6 |
| 79 | STM0830 | *glnH* | ABC superfamily (bind_prot), glutamine high-affinity transporter | 27244 | 139.2 | 53.2 |
| 80 | STM0154 | *lpdA* | lipoamide dehydrogenase (NADH); component of 2-oxodehydrogenase and pyruvate complexes; L protein of glycine cleavage complex second part | 50621 | 186.9 | 39.9 |
| 80-2 | STM1255 | *stm1255* | putative ABC transporter periplasmic binding protein | 60010 | 75.0 | 14.7 |
| 81 | STM2952 | *eno* | enolase | 45580 | 200.0 | 35.2 |
| 82 | STM1238 | *icdA* | isocitrate dehydrogenase in e14 prophage, specific for NADP+ | 45769 | 240.0 | 48.6 |

| | | | | | | |
|---|---|---|---|---|---|---|
| 83 | STM0981 | *rpsA* | 30S ribosomal subunit protein S1 | 61155 | 205.6 | 33.8 |
| 84 | STM4055 | *sodA* | superoxide dismutase, manganese | 23061 | 150.0 | 30.1 |
| 85 | STM2445 | *ucpA* | putative oxidoreductase | 27851 | 139.2 | 47.9 |
| 86 | STM0890 | *artI* | ABC superfamily (bind_prot), arginine transport system | 26978 | 74.7 | 22.6 |
| 87 | STM3287 | *nusA* | transcription pausing; L factor | 55407 | 156.4 | 25.8 |
| 88 | STM2786 | *stm2786* | tricarboxylic transport | 35461 | 102.9 | 22.5 |
| 89 | STM0133 | *ftsZ* | tubulin-like GTP-binding protein and GTPase, forms circumferential ring in cell division | 40305 | 178.6 | 48.6 |
| 90 | STM1431 | *sodB* | superoxide dismutase, iron | 21289 | 53.4 | 21.2 |
| 91 | STM2884 | *sipC* | cell invasion protein | 42965 | 158.3 | 42.1 |
| 92 | STM1478 | *ydgH* | putative periplasmic protein | 33897 | 177.8 | 59.2 |
| 93 | STM0219 | *frr* | ribosome releasing factor | 20505 | 90.5 | 35.1 |
| 94 | STM2444 | *cysP* | ABC superfamily (bind_prot), thiosulfate transport protein | 37564 | 233.9 | 61.8 |
| 95 | STM1586 | *stm1586* | putative periplasmic protein | 38485 | 130.8 | 32.0 |
| 96 | STM1700 | *fabI* | enoyl-[acyl-carrier-protein] reductase (NADH) | 27742 | 162.8 | 54.6 |
| 97 | STM0213 | *dapD* | 2,3,4,5-tetrahydropyridine-2-carboxylate N-succinyltransferase | 29834 | 198.6 | 44.2 |
| 98 | STM0232 | *accA* | acetylCoA carboxylase, carboxytransferase component, alpha subunit | 35325 | 75.5 | 29.2 |
| 99 | STM0242 | *proS* | proline tRNA synthetase | 63521 | 145.9 | 24.8 |
| 99-2 | STM0487 | *htpG* | chaperone Hsp90, heat shock protein C 62.5 | 72390 | 128.3 | 22.9 |
| 100 | STM3613 | *yhjJ* | putative Zn-dependent peptidase | 55162 | 207.7 | 40.6 |
| 101 | STM3446 | *fusA* | protein chain elongation factor EF-G, GTP-binding | 77581 | 284.5 | 44.6 |
| 102 | STM4330 | *mopA* | chaperone Hsp60 with peptide-dependent ATPase activity, affects cell division | 57267 | 192.4 | 24.3 |
| 103 | STM3445 | *tufA* | protein chain elongation factor EF-Tu (duplicate of tufB) | 43233 | 225.3 | 37.3 |
| 103-2 | STM4146 | *tufB* | protein chain elongation factor EF-Tu (duplicate of tufA) | 43265 | 212.0 | 37.3 |
| 104 | STM1117 | *agp* | glucose-1-phosphatase | 45540 | 159.1 | 27.6 |
| 105 | STM4561 | *osmY* | hyperosmotically inducible periplasmic protein, RpoS-dependent stationary phase gene | 21431 | 168.8 | 57.1 |
| 106 | STM3997 | *dsbA* | periplasmic protein disulfide isomerase I | 22893 | 132.4 | 45.4 |
| 106-2 | STM4561 | *osmY* | hyperosmotically inducible periplasmic protein, RpoS-dependent stationary phase gene | 21431 | 55.8 | 23.9 |
| 107 | STM0402 | *stm0402* | putative thiol - alkyl hydroperoxide reductase | 22299 | 150.8 | 62.0 |
| 108 | STM0435 | *yajQ* | putative cytoplasmic protein | 19003 | 113.2 | 41.4 |
| 108-2 | STM1440 | *sodC* | copper/zinc superoxide dismutase | 17719 | 64.8 | 33.5 |
| 109 | STM4055 | *sodA* | superoxide dismutase, manganese | 23061 | 133.3 | 37.4 |
| 110 | STM4249 | *aphA* | non-specific acid phosphatase/phosphotransferase, class B | 26296 | 98.7 | 40.5 |
| 111 | STM4081 | *tpiA* | triosephosphate isomerase | 26898 | 141.1 | 30.2 |
| 112 | STM0488 | *adk* | adenylate kinase | 23469 | 164.5 | 57.9 |
| 113 | STM1231 | *phoP* | response regulator in two-component regulatory system with PhoQ, transcribes genes expressed | 25615 | 107.4 | 42.0 |
| 113-2 | STM2355 | *argT* | ABC superfamily (bind_prot), lysine/arginine/ornithine transport protein | 28181 | 75.0 | 31.2 |
| 114 | STM0738 | *sucC* | succinyl-CoA synthetase, beta subunit | 41462 | 239.4 | 42.8 |
| 115 | STM3415 | *rpoA* | RNA polymerase, alpha subunit | 36493 | 186.9 | 48.6 |
| 116 | STM4184 | *aceA* | isocitrate lyase | 47544 | 139.6 | 29.7 |
| 117 | STM0152 | *aceE* | pyruvate dehydrogenase, decarboxylase component | 99561 | 164.8 | 14.9 |
| 118 | STM0737 | *sucB* | 2-oxoglutarate dehydrogenase (dihydrolipoyltranssuccinase E2 component) | 43839 | 197.9 | 38.3 |
| 119 | STM2660 | *clpB* | ATP-dependent protease, Hsp 100, part of novel multi-chaperone system with DnaK, DnaJ, and GrpE | 95418 | 264.1 | 34.3 |
| 120 | STM3380 | *accC* | acetyl CoA carboxylase, biotin carboxylase subunit | 49244 | 185.1 | 33.6 |
| 121 | STM0504 | *ybbN* | paral putative thioredoxin protein | 31796 | 96.8 | 25.4 |
| 122 | STM0007 | *talB* | transaldolase B | 35153 | 143.8 | 39.4 |
| 123 | STM3903 | *ilvE* | branched-chain amino-acid aminotransferase | 34034 | 84.9 | 23.9 |
| 124 | STM1599 | *pdgL* | periplasmic dipeptidase for D-ala-D-ala digestion in peptidoglycan | 28977 | 64.4 | 17.2 |
| 125 | STM1119 | *wraB* | trp-repressor binding protein | 20849 | 96.5 | 44.4 |
| 126 | STM3472 | *ppiA* | peptidyl-prolyl cis-trans isomerase A (rotamase A) | 20314 | 72.9 | 22.1 |
| 127 | STM1150 | *mdoG* | periplasmic glucans biosynthesis protein | 57826 | 108.6 | 25.2 |
| 127-2 | STM1679 | *mppA* | periplasmic murein tripeptide transport protein, also negative regulator of mulitple antibiotic | 59859 | 95.6 | 22.2 |
| 128 | STM2259 | *napA* | periplasmic nitrate reductase, large subunit, in complex with NapB | 92855 | 160.6 | 20.3 |
| 129 | STM4242 | *stm4242* | putative outer membrane or exported | 46927 | 54.5 | 16.1 |
| 129-2 | STM3188 | *ygiC* | putative glutathionylspermidine synthase | 44962 | 38.2 | 8.0 |
| 130 | STM2267 | *ompC* | outer membrane protein 1b (ib;c), porin | 41220 | 78.8 | 17.2 |
| 131 | STM1572 | *nmpC* | new outer membrane protein; predicted bacterial porin | 39661 | 55.0 | 13.8 |
| 132 | STM4077 | *yneA* | putative ABC superfamily (peri_perm), sugar transport protein | 36741 | 182.7 | 56.5 |
| 133 | STM2861 | *sitA* | Salmonella iron transporter: fur regulated | 33704 | 164.8 | 43.6 |
| 134 | STM0216 | *rpsB* | 30S ribosomal subunit protein S2 | 26740 | 135.8 | 34.9 |
| 135 | STM0735 | *sdhB* | succinate dehydrogenase, Fe-S protein | 26846 | 146.2 | 55.2 |
| 136 | STM3968 | *udp* | uridine phosphorylase | 27121 | 106.8 | 34.4 |
| 137 | STM4342 | *frdB* | fumarate reductase, anaerobic, Fe-S protein subunit | 27157 | 129.4 | 42.6 |
| 138 | STM3043 | *dsbC* | protein disulfide isomerase II | 25817 | 128.5 | 49.4 |
| 139 | STM4055 | *sodA* | superoxide dismutase, manganese | 23061 | 75.1 | 33.0 |
| 139-2 | STM1884 | *eda* | multifunctional:2-keto-3-deoxygluconate 6-phosphate aldolase; 2-keto-4-hydroxyglutarate aldolase; oxaloacetate decarboxylase | 22168 | 71.7 | 25.4 |
| 139-3 | STM1451 | *gst* | glutathionine S-transferase | 22424 | 64.7 | 35.8 |
| 140 | STM2433 | *crr* | PTS family, glucose-specific IIA component | 18229 | 108.0 | 46.2 |
| 141 | STM0608 | *ahpC* | alkyl hydroperoxide reductase, C22 subunit; detoxification of hydroperoxides | 20729 | 135.1 | 50.8 |
| 142 | STM3063 | *rpiA* | ribosephosphate isomerase, constitutive | 22877 | 53.3 | 22.8 |
| 142-2 | STM4334 | *efp* | elongation factor P (EF-P) | 20605 | 40.5 | 21.8 |
| 143 | STM2498 | *upp* | uracil phosphoribosyltransferase | 22515 | 106.8 | 45.7 |
| 144 | STM0578 | *nfnB* | dihydropteridine reductase/oxygen-insensitive NAD(P)H nitroreductase | 23937 | 114.6 | 46.5 |
| 145 | STM4190 | *pepE* | (alpha)-aspartyl dipeptidase | 24751 | 76.3 | 34.9 |
| 146 | STM1690 | *pspA* | phage shock protein; negative regulatory gene for the psp opreon | 25505 | 99.5 | 39.6 |
| 146-2 | STM0711 | *stm0711* | putative cytoplasmic protein | 26927 | 43.5 | 25.5 |
| 147 | STM1729 | *yciF* | putative cytoplasmic protein | 18634 | 105.7 | 43.1 |
| 148 | STM3551 | *ggt* | gamma-glutamyltranspeptidase | 61702 | 58.8 | 11.2 |
| 149 | STM1251 | *stm1251* | putative molecular chaperone (small heat shock protein) | 17532 | 86.7 | 52.3 |
| 150 | STM2817 | *luxS* | quorum sensing protein, produces autoinducer - acyl-homoserine lactone-signaling molecules | 19290 | 54.3 | 22.8 |
| 151 | STM0961 | *lolA* | periplasmic protein effects translocation of lipoproteins from inner membrane to outer membrane | 22594 | 107.2 | 61.8 |
| 152 | STM1070 | *ompA* | putative hydrogenase, membrane component | 37497 | 148.7 | 21.1 |
| 153 | STM3318 | *yhbN* | putative ABC superfamily (bind_prot) transport protein | 20040 | 128.8 | 52.2 |
| 154 | STM4405 | *ytfJ* | putative transcriptional regulator | 20558 | 168.4 | 37.3 |
| 155 | STM0734 | *sdhA* | succinate dehydrogenase, flavoprotein subunit | 64443 | 182.6 | 29.3 |
| 156 | STM3500 | *pckA* | phosphoenolpyruvate carboxykinase | 59558 | 211.4 | 40.1 |
| 157 | STM0421 | *yajO* | putative oxidoreductase / K + channel protein | 36143 | 170.9 | 41.7 |
| 158 | STM3090 | *metK* | methionine adenosyltransferase 1 (AdoMet synthetase) | 41934 | 107.1 | 26.3 |
| 159 | STM0785 | *ybhE* | putative 3-carboxymuconate cyclase | 36318 | 86.7 | 23.3 |
| 160 | STM3348 | *degQ* | serine endoprotease | 47308 | 187.4 | 35.4 |
| 161 | STM1310 | *nadE* | NAD synthetase, prefers NH3 over glutamine | 30465 | 115.2 | 29.5 |
| 162 | STM0186 | *dksA* | dnaK suppressor protein | 17497 | 54.7 | 17.9 |
| 163 | STM1682 | *tpx* | thiol peroxidase | 18007 | 94.1 | 23.8 |
| 164 | STM0536 | *ppiB* | peptidyl-prolyl cis-trans isomerase B (rotamase B) | 18122 | 64.8 | 52.4 |
| 165 | STM0739 | *sucD* | succinyl-CoA synthetase, alpha subunit | 29757 | 117.5 | 29.1 |
| 165-2 | STM1772 | *kdsA* | 3-deoxy-D-manno-octulosonic acid 8-P synthetase | 30777 | 80.8 | 20.1 |
| 166 | STM4078 | *yneB* | putative fructose-1,6-bisphosphate aldolase | 31724 | 127.9 | 40.9 |
| 167 | STM3359 | *mdh* | malate dehydrogenase | 32457 | 115.8 | 36.5 |
| 167-2 | STM2430 | *cysK* | subunit of cysteine synthase A and O-acetylserine sulfhydrolase A | 34517 | 113.9 | 43.0 |

| | | | | | | |
|---|---|---|---|---|---|---|
| 168 | STM0076 | *fixB* | putative electron transfer flavoprotein, carnitine metabolism | 37115 | 104.4 | 26.6 |
| 168-2 | STM2016 | *cobT* | nicotinate-nucleotide dimethylbenzimidazole-P phophoribosyl transferase | 36594 | 32.1 | 8.4 |
| 169 | STM1163 | *pyrC* | dihydro-orotase | 38587 | 72.7 | 20.1 |
| 170 | STM4062 | *pfkA* | 6-phosphofructokinase I | 34897 | 125.1 | 35.6 |
| 171 | STM2555 | *glyA* | serine hydroxymethyltransferase | 45436 | 116.0 | 24.5 |
| 172 | STM4086 | *glpK* | glycerol kinase | 56045 | 188.1 | 36.9 |
| 173 | STM4091 | *hslU* | ATPase component of the HslUV protease | 49649 | 187.7 | 37.7 |
| 174 | STM3710 | *rfaD* | ADP-L-glycero-D-mannoheptose-6-epimerase | 34831 | 228.9 | 41.9 |
| 175 | STM3374 | *mreB* | rod shape-determining protein; HSP70 class molecular chaperones involved in cell morphogenesis | 36934 | 137.1 | 37.8 |
| 176 | STM1752 | *galU* | glucose-1-phosphate uridylyltransferase | 32888 | 87.1 | 21.5 |
| 177 | STM0372 | *hemB* | 5-aminolevulinate dehydratase ( porphobilinogen synthase) | 35529 | 64.7 | 20.7 |
| 178 | STM3069 | *pgk* | phosphoglycerate kinase | 41114 | 84.9 | 18.6 |
| 179 | STM0773 | *galM* | galactose-1-epimerase (mutarotase) | 38497 | 75.1 | 19.7 |
| 180 | STM1337 | *pheS* | phenylalanine tRNA synthetase, alpha-subunit | 36736 | 92.2 | 23.9 |
| 180-2 | STM3708 | *tdh* | threonine 3-dehydrogenase | 37194 | 73.0 | 17.6 |
| 181 | STM2578 | *pdxJ* | carries out condensation and ring closure step after PdxA in pyridoxine biosynthesis | 26325 | 82.6 | 32.9 |
| 182 | STM1222 | *potD* | ABC superfamily (peri_perm), spermidine/putrescine transporter | 39003 | 158.3 | 39.1 |
| 183 | STM3557 | *ugpB* | ABC superfamily (peri_perm), sn-glycerol 3-phosphate transport protein | 48374 | 81.2 | 21.2 |
| 183-2 | STM0748 | *tolB* | tol protein required for outer membrane integrity, uptake of group A colicins, and translocation of phage DNA to cytoplasm, may be part of multiprotein peptidoglycan recycling complex (Two domains) | 46130 | 53.2 | 13.3 |
| 184 | STM3348 | *degQ* | serine endoprotease | 47308 | 136.4 | 24.2 |
| 185 | STM2061 | *sbmC* | DNA gyrase inhibitor | 18044 | 50.8 | 24.5 |
| 186 | STM3282 | *pnp* | polynucleotide phosphorylase, member of mRNA degradosome | 77002 | 140.6 | 21.2 |
| 187 | STM3446 | *fusA* | protein chain elongation factor EF-G, GTP-binding | 77581 | 125.8 | 20.2 |
| 188 | STM0158 | *acnB* | aconitate hydratase 2 | 93479 | 147.9 | 16.8 |
| 189 | STM0153 | *aceF* | pyruvate dehydrogenase, dihydrolipoyltransacetylase component | 66122 | 177.4 | 21.3 |
| 190 | STM0228 | *lpxA* | UDP-N-acetylglucosamine acetyltransferase | 28038 | 84.5 | 29.8 |
| 191 | STM3868 | *atpH* | membrane-bound ATP synthase, F1 sector, delta-subunit | 19394 | 49.1 | 23.2 |
| 191-2 | STM4561 | *osmY* | hyperosmotically inducible periplasmic protein, RpoS-dependent stationary phase gene | 21431 | 46.7 | 15.6 |
| 192 | STM0803 | *moaB* | molybdopterin biosynthesis, protein B | 18521 | 97.0 | 34.7 |
| 193 | STM3483 | *rpe* | D-ribulose-5-phosphate 3-epimerase | 24473 | 39.4 | 6.7 |
| 193-2 | STM0171 | *yadF* | putative carbonic anhydrase | 24803 | 31.5 | 7.7 |
| 194 | STM1720 | *yciO* | putative translation factor | 23088 | 32.1 | 11.2 |
| 195 | STM3165 | *yqhE* | 2,5-diketo-D-gluconate reductase A | 30977 | 115.9 | 21.8 |
| 196 | STM1493 | *stm1493* | putative periplasmic component, ABC transport system | 33865 | 113.3 | 25.3 |
| 196-2 | STM1599 | *pdgL* | Periplasmic dipeptidase for D-ala-D-ala digestion in peptidoglycan | 28977 | 42.3 | 16.0 |
| 197 | STM0684 | *nagB* | glucosamine-6-phosphate deaminase | 29613 | 43.2 | 18.0 |
| 198 | STM1302 | *xthA* | exonuclease III, may repair singlet oxygen induced lesions | 30767 | 111.0 | 31.7 |
| 199 | STM4404 | *cysQ* | affects pool of 3'-phosphoadenosine-5'-phosphosulfate in pathway of sulfite synthesis | 27472 | 85.5 | 28.5 |
| 199-2 | STM1358 | *aroD* | 3-dehydroquinate dehydratase | 27307 | 53.9 | 22.2 |
| 200 | STM4598 | *arcA* | response regulator (OmpR family) in two-component regulatory system with ArcB (or CpxA), regulates genes in aerobic pathways | 27273 | 105.1 | 21.0 |
| 201 | STM1731 | *stm1731* | putative catalase | 31828 | 74.6 | 19.5 |
| 201-2 | STM1970 | *fliG* | flagellar biosynthesis, component of motor switching and energizing | 36833 | 31.8 | 5.1 |
| 202 | STM2883 | *sipD* | cell invasion protein | 37094 | 95.8 | 28.3 |
| 202-2 | STM0145 | *nadC* | quinolinate phosphoribosyltransferase | 32541 | 40.1 | 11.1 |
| 203 | STM1953 | *yedO* | putative 1-cyclopropane-carboxylate deaminase | 34893 | 115.1 | 24.7 |
| 203-2 | STM0958 | *trxB* | thioredoxin reductase | 34666 | 54.2 | 16.1 |
| 204 | STM1070 | *ompA* | putative hydrogenase, membrane component | 37497 | 136.9 | 35.4 |
| 205 | STM3539 | *asd* | aspartate-semialdehyde dehydrogenase | 40118 | 62.6 | 8.4 |
| 206 | STM3164 | *yqhD* | putative alcohol dehydrogenase | 42065 | 75.0 | 17.6 |
| 206-2 | STM1976 | *fliM* | flagellar biosynthesis, component of motor switch and energizing | 37840 | 41.7 | 11.1 |
| 207 | STM3339 | *nanA* | N-acetylneuraminate lyase (aldolase) | 32437 | 85.8 | 18.2 |
| 207-2 | STM2307 | *menB* | dihydroxynaphtoic acid synthetase | 31675 | 52.5 | 15.1 |
| 208 | STM1328 | *stm1328* | putative outer membrane protein | 34789 | 74.4 | 13.8 |
| 208-2 | STM1700 | *fabI* | enoyl-[acyl-carrier-protein] reductase (NADH) | 27742 | 48.6 | 16.4 |
| 209 | STM1960 | *fliD* | flagellar biosynthesis; filament capping protein; enables filament assembly | 49816 | 181.3 | 33.0 |
| 209-2 | STM3597 | *gor* | glutathione oxidoreductase | 48674 | 71.9 | 10.2 |
| 210 | STM1305 | *astD* | succinylglutamic semialdehyde dehydrogenase | 52999 | 43.2 | 9.3 |
| 211 | STM4077 | *yneA* | putative ABC superfamily (peri_perm), sugar transport protein | 36741 | 86.2 | 24.1 |
| 211-2 | STM4460 | *pyrB* | aspartate carbamoyltransferase, catalytic subunit | 34366 | 74.7 | 20.9 |
| 212 | STM1290 | *gapA* | glyceraldehyde-3-phosphate dehydrogenase A | 35568 | 153.6 | 28.1 |
| 213 | STM1796 | *treA* | trehalase, periplasmic | 63491 | 74.2 | 10.5 |
| 214 | STM0494 | *ushA* | UDP-sugar hydrolase 5'-nucleotidase | 60538 | 97.5 | 12.4 |
| 214-1 | STM0683 | *nagA* | N-acetylglucosamine-6-phosphate deacetylase | 41092 | 53.9 | 14.6 |
| 215 | STM2487 | *purC* | phosphoribosylaminoimidazole-succinocarboxamide synthetase (SAICAR synthetase) | 26889 | 63.6 | 13.9 |
| 216 | STM3063 | *rpiA* | ribosephosphate isomerase, constitutive | 22877 | 67.0 | 11.0 |
| 217 | STM2335 | *yfbU* | putative cytoplasmic protein | 19506 | 103.7 | 37.8 |
| 218 | STM1567 | *adhP* | alcohol dehydrogenase, propanol preferring | 35522 | 80.9 | 18.2 |
| 219 | STM2354 | *hisJ* | ABC superfamily (bind_prot), histidine transport protein | 28361 | 111.5 | 22.3 |
| 220 | STM2433 | *crr* | PTS family, glucose-specific IIA component | 18229 | 42.8 | 24.3 |
| 221 | STM3487 | *aroK* | shikimate kinase I | 19451 | 74.3 | 42.2 |
| 222 | STM3502 | *ompR* | response regulator in two-component regulatory system with EnvZ, affecting transcription of ompC and ompF (OmpR family) | 27335 | 83.8 | 30.1 |
| 223 | STM2211 | *yeiP* | putative elongation factor | 29925 | 69.9 | 18.0 |
| 224 | STM1157 | *yceI* | putative secreted protein | 21004 | 52.9 | 21.5 |
| 225 | STM2262 | *eco* | ecotin, a serine protease inhibitor | 18199 | 37.6 | 13.4 |
| 226 | STM3445 | *tufA* | protein chain elongation factor EF-Tu (duplicate of tufB) | 43233 | 121.8 | 19.5 |
| 226-2 | STM4146 | *tufB* | protein chain elongation factor EF-Tu (duplicate of tufA) | 43265 | 116.0 | 19.5 |
| 227 | STM2378 | *fabB* | 3-oxoacyl-[acyl-carrier-protein] synthase I | 42355 | 52.7 | 10.9 |
| 228 | STM0310 | *ghmA* | phosphoheptose isomerase | 20877 | 80.7 | 28.6 |
| 229 | STM4561 | *osmY* | hyperosmotically inducible periplasmic protein, RpoS-dependent stationary phase gene | 21431 | 31.6 | 9.8 |
| 230 | STM1730 | *yciE* | putative cytoplasmic protein | 18954 | 21.1 | 4.8 |
| 231 | STM1746 | *oppA* | ABC superfamily (periplasm), oligopeptide transport protein with chaperone properties | 65517 | 184.4 | 20.4 |
| 232 | STM0653 | *ybeL* | putative cytoplasmic protein | 18392 | 58.4 | 17.2 |
| 233 | STM4152 | *rplL* | 50S ribosomal subunit protein L7/L12 | 12281 | 74.9 | 27.3 |
| 234 | STM4152 | *rplL* | 50S ribosomal subunit protein L7/L12 | 12281 | 63.5 | 36.4 |
| 235 | STM4152 | *rplL* | 50S ribosomal subunit protein L7/L12 | 12281 | 52.5 | 36.4 |
| 236 | STM3591 | *uspA* | universal stress protein A | 16062 | 23.5 | 16.7 |
| 237 | STM3591 | *uspA* | universal stress protein A | 16062 | 32.4 | 16.7 |
| 238 | STM4391 | *rpsF* | 30S ribosomal subunit protein S6 | 15154 | 32.2 | 15.3 |
| 239 | STM1751 | *hns* | DNA-binding protein HLP-II (HU, BH2, HD, NS); pleiotropic regulator | 15524 | 63.0 | 29.9 |
| 240 | STM4329 | *mopB* | chaperone Hsp10, affects cell division | 10299 | 61.4 | 53.6 |
| 241 | STM4329 | *mopB* | chaperone Hsp10, affects cell division | 10299 | 60.7 | 53.6 |
| 242 | STM1652 | *ynaF* | putative universal stress protein | 15696 | 71.4 | 23.6 |
| 243 | STM4391 | *rpsF* | 30S ribosomal subunit protein S6 | 15154 | 63.7 | 23.7 |
| 243-2 | STM1652 | *ynaF* | putative universal stress protein | 15696 | 41.8 | 13.2 |
| 244 | STM0211 | *yaeH* | putative cytoplasmic protein | 15076 | 142.0 | 78.9 |
| 245 | STM0211 | *yaeH* | putative cytoplasmic protein | 15076 | 142.0 | 78.9 |

| | | | | | | |
|---|---|---|---|---|---|---|
| 246 | STM4394 | *rplI* | 50S ribosomal subunit protein L9 | 15766 | 115.3 | 56.4 |
| 247 | STM3439 | *rplD* | 50S ribosomal subunit protein L4, regulates expression of S10 operon | 22068 | 60.6 | 14.9 |
| 247-2 | STM1195 | *fabG* | 3-oxoacyl-[acyl-carrier-protein] reductase | 25527 | 31.5 | 11.9 |
| 248 | STM3439 | *rplD* | 50S ribosomal subunit protein L4, regulates expression of S10 operon | 22068 | 60.3 | 19.4 |
| 248-2 | STM3310 | *yrbC* | putative ABC superfamily (atp&memb), transport protein | 23986 | 43.9 | 12.8 |
| 249 | STM2795 | *ygaU* | putative LysM domain | 16103 | 68.6 | 41.6 |
| 250 | STM1044 | *sodC* | Gifsy-2 prophage: superoxide dismutase precursor (Cu-Zn) | 18352 | 44.4 | 14.7 |
| 251 | STM4458 | *yjgF* | putative translation initiation inhibitor | 13557 | 60.9 | 25.0 |
| 251-2 | STM2646 | *yfiD* | putative formate acetyltransferase | 14326 | 52.8 | 25.2 |
| 252 | STM2646 | *yfiD* | putative formate acetyltransferase | 14326 | 84.2 | 33.9 |
| 252-2 | STM4458 | *yjgF* | putative translation initiation inhibitor | 13557 | 61.3 | 25.0 |
| 253 | STM2646 | *yfiD* | putative formate acetyltransferase | 14326 | 122.3 | 53.5 |
| 254 | STM0417 | *ribH* | riboflavin synthase, beta chain | 15990 | 21.5 | 15.4 |
| 255 | STM1563 | *osmC* | putative resistance protein, osmotically inducible | 15084 | 42.0 | 14.0 |
| 256 | STM0653 | *ybeL* | putative cytoplasmic protein | 18392 | 42.3 | 14.6 |
| 257 | STM3915 | *trxA* | thioredoxin 1, redox factor | 11788 | 29.3 | 11.0 |
| 258 | STM2526 | *ndk* | nucleoside diphosphate kinase | 15503 | 43.3 | 33.6 |
| 259 | STM2526 | *ndk* | nucleoside diphosphate kinase | 15503 | 37.4 | 19.6 |
| 260 | STM2262 | *eco* | ecotin, a serine protease inhibitor | 13876 | 31.7 | 11.5 |
| 261 | STM0317 | *gpt* | guanine-hypoxanthine phosphoribosyltransferase | 16951 | 37.5 | 11.8 |
| 261-2 | STM2262 | *eco* | ecotin, a serine protease inhibitor | 18199 | 37.5 | 8.5 |
| 262 | STM4151 | *rplJ* | 50S ribosomal subunit protein L10 | 17782 | 32.6 | 4.2 |
| 263 | STM3176 | *ygiW* | putative outer membrane protein | 13926 | 29.1 | 8.5 |
| 264 | STM0803 | *moaB* | molybdopterin biosynthesis, protein B | 18521 | 45.9 | 15.3 |
| 264-2 | STM4561 | *osmY* | hyperosmotically inducible periplasmic protein, RpoS-dependent stationary phase gene | 21431 | 21.1 | 12.7 |
| 265 | STM3318 | *yhbN* | putative ABC superfamily (bind_prot) transport protein | 20040 | 57.3 | 17.4 |
| 266 | STM3426 | *rpsH* | 30S ribosomal subunit protein S8, and regulator | 14108 | 33.9 | 23.1 |
| 267 | STM0225 | *hlpA* | histone-like protein, located in outer membrane | 17855 | 21.2 | 13.7 |
| 268 | STM1855 | *sopE2* | TypeIII-secreted protein effector: invasion-associated protein | 26398 | 83.9 | 22.5 |
| 269 | STM4405 | *ytfJ* | putative transcriptional regulator | 20558 | 68.9 | 15.1 |
| 270 | STM4405 | *ytfJ* | putative transcriptional regulator | 20558 | 62.9 | 24.3 |
| 271 | STM0959 | *lrp* | regulator for lrp regulon and high-affinity branched-chain amino acid transport system; mediator of of leucine response (AsnC family) | 18838 | 123.9 | 40.9 |
| 272 | STM4587 | *creA* | putative periplasmic protein | 17184 | 81.7 | 31.8 |
| 273 | STM3701 | *secB* | molecular chaperone in protein export | 17227 | 21.3 | 14.2 |
| 274 | STM0366 | *yahO* | putative periplasmic protein | 9901 | 21.7 | 23.1 |
| 275 | STM0219 | *frr* | ribosome releasing factor | 20505 | 63.8 | 23.2 |
| 276 | STM1682 | *tpx* | thiol peroxidase | 18007 | 38.1 | 14.9 |
| 276-2 | STM1730 | *yciE* | putative cytoplasmic protein | 18954 | 21.1 | 4.8 |
| 277 | STM3176 | *ygiW* | putative outer membrane protein | 13926 | 37.8 | 19.2 |
| 277-2 | STM3108 | *yggL* | putative cytoplasmic protein | 12776 | 21.2 | 24.1 |
| 278 | STM0694 | *fldA* | flavodoxin 1 | 16918 | 40.0 | 11.4 |
| 279 | STM1196 | *acpP* | acyl carrier protein | 8621 | 41.9 | 12.8 |
| 280 | STM1196 | *acpP* | acyl carrier protein | 8621 | 42.0 | 12.8 |
| 281 | STM1196 | *acpP* | acyl carrier protein | 8621 | 31.1 | 12.8 |
| 282 | STM1431 | *sodB* | superoxide dismutase, iron | 21289 | 31.7 | 8.3 |
| 283 | STM2061 | *sbmC* | DNA gyrase inhibitor | 18044 | 81.6 | 24.5 |
| 284 | STM2433 | *crr* | PTS family, glucose-specific IIA component | 18229 | 64.2 | 23.1 |
| 285 | STM2433 | *crr* | PTS family, glucose-specific IIA component | 18229 | 84.6 | 43.8 |
| 286 | STM3379 | *accB* | acetylCoA carboxylase, BCCP subunit, carrier of biotin | 16669 | 51.0 | 30.8 |
| 287 | STM1070 | *ompA* | putative hydrogenase, membrane component | 37497 | 120.8 | 18.6 |
| 288 | STM0961 | *lolA* | periplasmic protein effects translocation of lipoproteins from inner membrane to outer membrane | 22594 | 72.0 | 25.0 |
| 289 | STM4148 | *nusG* | component in transcription antitermination | 20527 | 49.8 | 17.1 |
| 289-2 | STM1210 | *ycfP* | putative esterase | 21059 | 21.6 | 13.9 |
| 290 | STM3428 | *rplE* | 50S ribosomal subunit protein L5 | 20299 | 88.9 | 31.8 |
| 291 | STM3428 | *rplE* | 50S ribosomal subunit protein L5 | 20299 | 71.7 | 12.0 |
| 292 | STM1920 | *cheW* | purine-binding chemotaxis protein; regulation | 18030 | 71.5 | 27.5 |
| 293 | STM3915 | *trxA* | thioredoxin 1, redox factor | 11788 | 31.6 | 26.6 |
| 294 | STM1118 | *yccJ* | putative cytoplasmic protein | 13876 | 21.1 | 17.7 |
| 295 | STM0186 | *dksA* | dnaK suppressor protein | 17497 | 52.7 | 34.4 |
| 296 | STM0435 | *yajQ* | putative cytoplasmic protein | 19003 | 113.9 | 42.0 |
| 297 | STM1751 | *hns* | DNA-binding protein HLP-II (HU, BH2, HD, NS); pleiotropic regulator | 15524 | 71.9 | 29.2 |
| 297-2 | STM2491 | *bcp* | thiol peroxidase, thioredoxin dependent | 17590 | 53.9 | 25.0 |
| 298 | STM2491 | *bcp* | thiol peroxidase, thioredoxin dependent | 17590 | 80.4 | 30.1 |
| 299 | STM3487 | *aroK* | shikimate kinase I | 19451 | 168.9 | 75.7 |
| 300 | STM1652 | *ynaF* | putative universal stress protein | 15696 | 52.9 | 18.8 |
| 301 | STM4391 | *rpsF* | 30S ribosomal subunit protein S6 | 15154 | 64.1 | 23.7 |
| 302 | STM4151 | *rplJ* | 50S ribosomal subunit protein L10 | 16028 | 31.9 | 30.8 |
| 303 | STM1837 | *cspC* | cold shock protein, multicopy suppresses mukB mutants, putative regulator | 7384 | 52.1 | 62.3 |
| 304 | STM3864 | *atpC* | membrane-bound ATP synthase, F1 sector, epsilon-subunit | 15046 | 31.4 | 5.8 |
| 305 | STM1157 | *yceI* | putative secreted protein | 21004 | 71.9 | 25.7 |
| 306 | STM0506 | *tesA* | multifunctional acyl-CoA thioesterase I; also functions as protease I, lysophospholipaseL(I) | 23033 | 84.9 | 27.5 |
| 307 | STM2799 | *stpA* | DNA-binding protein with chaperone activity | 15469 | 68.7 | 23.3 |
| 307-2 | STM4149 | *rplK* | 50 S ribosomal subunit protein L11 | 14857 | 42.8 | 12.7 |

[a]Theoritical molecular weight calculated from the amino acid sequence.
[b]The highest score of SEQUEST search in the most likely candidate.
[c]Accordance rate of peptides confirmed by MS/MS analyses in amino acid sequences of candidate proteins
